# IL-33 ameliorates experimental colitis involving regulation of autophagy of macrophages in mice

**DOI:** 10.1186/s13578-019-0271-5

**Published:** 2019-01-14

**Authors:** Zhongyan Wang, Lifeng Shi, Shuyao Hua, Chang Qi, Min Fang

**Affiliations:** Department of Immunology, School of Basic Medicine, Tongji Medical College, Huazhong University of Science and Technology, 13# Hangkong Road, Wuhan, China

**Keywords:** IL-33, TNBS-induced experimental colitis, Autophagy, Macrophages, TLR4

## Abstract

**Background:**

Previously, we have demonstrated that IL-33 administration protecting TNBS-induced experimental colitis is associated with facilitation of Th2/Tregs responses in mice. However, whether IL-33 regulates autophagy to ameliorate experimental colitis is unclear.

**Results:**

IL-33 administration (2 μg/day, intraperitoneal injection), while facilitating Th2/Tregs responses, also enhances the autophagy in mice with TNBS-induced colitis as well as macrophages. In the meantime, we observed that inhibition of the autophagy with 3-methyladenine (3-MA) (24 mg/kg, intraperitoneal injection) in mice exacerbates TNBS-induced experimental colitis. On the contrary, administration of rapamycin (2 mg/kg,intragastric administration), an autophagy-enhancer, alleviates the colitis in mice. In vivo, Immunofluorescence analysis revealed that TNBS combined with IL-33 enhanced the autophagy of macrophages in the inflammatory gut tissue. In vitro, treatment with IL-33 promoted the autophagy of macrophages generated from bone marrow cells in dose-dependant manner. Furthermore, the effect of autophagy-enhancement by IL-33 is TLR4 signaling pathway dependant. Our notion was further confirmed by IL-33-deficient bone marrow-derived macrophages cells.

**Conclusions:**

IL-33 regulates the autophagy is a new immunoregulatory property on TNBS-induced experimental colitis in mice.

## Background

Interleukin (IL)-33 is a member of the IL-1 family of cytokines. IL-33 is a nuclear protein that is also released into the extracellular space, and thus acts as a dual-function molecule, as does IL-1α. Extracellular IL-33 binds to the cell-surface receptor ST2, leading to the activation of intracellular signaling pathways similar to those used by IL-1. Unlike conventional cytokines, IL-33 might be secreted via unconventional pathways, and can be released upon cell injury as an alarmin [1]. Moreover, like HMGB1 (high-mobility group box 1), IL-33 has been suggested to act as an “alarmin” that amplifies immune responses during tissue injury. In contrast to HMGB1, however, the precise roles of IL-33 in those settings are poorly understood [2, 3].

Autophagy is an evolutionarily conserved lysosomal mechanism that enables cells to conserve and maintain cellular biomass quality and quantity by targeting damaged or unused proteins and even organelles for degradation [4–6]. Previously, we and others have demonstrated that IL-33 ameliorates experimental colitis through promoting Th2/Foxp3 + regulatory T Cell responses in the experimental colitis in mice [7–10], and IL-33 expression actually increased in the inflamed mucosa of IBD patients, in particular in UC patients. Furthermore, colonic subepithelial myofibroblasts (SEMFs) is a source of IL-33 in the human colonic mucosa [11]. However, whether IL-33 ameliorates experimental colitis through regulating autophagy is unknown. In this study, we show that TNBS-induced colitis in mice was exacerbated when autophagy was inhibited by 3-MA. On the contrary, administration of TNBS combined with rapamycin, an autophagy-enhancer, the mice displayed less severe colitis as compared with treatment with TNBS alone. Similarly, we demonstrate that IL-33 ameliorates TNBS-induced colitis via enhancing the autophagy. Moreover, IL-33 enhances the autophagy via regulates the TLR4 signaling pathway.

## Materials and methods

### Mice

Male BALB/c mice of 6–8 weeks old were purchased from the Institute of Experimental Animal, Chinese Academy of Medical Sciences (Beijing, China). Male C57/BL6 mice 5–6 weeks of age were purchased from Centers for Disease Control (CDC), Wuhan (Hubei, China). C57BL/10^ScNJNju^ (TLR4^−/−^) mice were purchased from biomedical research institute (Nanjing, China). IL-33 gene knockout mice with C57BL/6 background were obtained from the laboratory of Dr. Fang Zheng in Department of Immunology, Tongji Medical College, HUST (China). The mice were housed in the SPF facility at the Tongji Medical College for at least 1 week before inclusion in experiments. All of the studies were performed in accordance with the Tongji Medical College Animal Care and Use Committee guidelines.

### Reagents and antibodies

2,4,6-trinitrobenzenesulfonic acid (TNBS), 3-methyladenine (3-MA), starvation medium EBSS (Earle’s balanced salt solution), chloroquine and rapamycin were purchased from Sigma-Aldrich (St. Louis, MO, USA). Mouse IL-33 was purchased from Pepro Tech Inc (London, UK). Antibodies against Beclin-1, LC3B I/II, P62 and β-actin were purchased from Sigma-Aldrich (St. Louis, MO, USA).

### Induction of TNBS-colitis and treatment with 3-MA or rapamycin

Colitis was induced by TNBS in male BALB/c mice as described elsewhere [12]. In brief, 2.5 mg of TNBS (Sigma-Aldrich Corp., St. Louis, MO) dissolved in 50% ethanol (EtoH) (total volume, 100 μl) was administered intrarectally to lightly anesthetized (methoxyflurane) mice through a polyurethane catheter (Becton–Dickinson, San Jose, CA, USA) equipped with a 1-ml syringe. Control mice received 50% EtoH using the same technique. The body weight and disease activity of each mouse were evaluated every day after TNBS or EtoH administration. All mice were euthanized on 4th day after induction of colitis. Mice were randomly assigned to five groups: the EtoH + PBS group, EtoH + TNBS group, TNBS + 3-MA group (24 mg/kg, intraperitoneal injection), TNBS + IL-33 (2 μg/mouse/day, intraperitoneal injection) and TNBS + Rapamycin group (2 mg/kg,intragastric administration). Each group was consisted of 3–6 mice.

### Histological analysis

The mice were sacrificed by cervical dislocation. Macroscopic assessment of inflammation was scored as described in previous study [12]. Subsequently, samples of colon tissues were prepared for tissue sections, and then stained with hematoxylin and eosin. Histological analysis was performed as described in previous report [13].

### Immunofluorescence staining

The formation of autophagosomes in colonic macrophages after TNBS induction with or without IL-33 administration was compared by immunofluorescence assay. Briefly, the sections were deparaffinized, rehydrated and washed in 1% PBS Tween. Then they were treated with 3% hydrogen peroxide, blocked with 5% bovine serum albumin (BSA) and incubated simultaneously with Beclin-1 (1:500, Sigma) and F4/80 (1:500, Abcam) overnight. After staining with the- secondary antibody, the slides were then counter-stained with DAPI for 5 min. Images were acquired by a fluorescence microscope (Olympus, Lake Success, NY). Settings for image acquisition were identical for control and experimental tissues.

### Generation of mouse BMs and incubated with IL-33, IL-33 plus chloroquine as well as rapamycin respectively

Mouse bone marrow derived macrophages (BMs) were propagated from bone marrow cells as described previously [14]. Recombinant cytokine M-CSF in vitro experiments were obtained from Peprotech (London, UK). BMs were incubated with IL-33 (100 ng/ml) for 24 h. Experimental groups were designed as follows: 1) PBS control group, 2) IL-33 alone group, 3) IL-33 combined with chloroquine (50 μM). Thereafter, the proteins were extracted for western blotting analysis. Macrophages were treated with rapamycin (500 ng/ml) for 24 h. Thereafter, the cells were lysed for western blot examination.

### Western blotting

Colonic samples were homogenized in ice-cold lysis buffer with a protease inhibitor cocktail (Sigma-Aldrich Corp., St. Louis, MO). Specimens were then purified by centrifugation at 10,000*g* for 10 min at 4 °C. Proteins were separated by 10% sodium dodecyl sulfate polyacrylamide gel electrophoresis (SDS-PAGE) gels and transferred to polyvinylidene fluoride (PVDF) membrane ((EMD Millipore, Billerica, MA). The membrane was incubated overnight in blocking buffer with primary LC3BI/II,P62, Beclin-1 and β-actin antibodies at 4 °C.

### Statistical analysis

The data are presented as mean ± SD. Statistical differences were determined by Student’s t test. Two-sided probability (p) values less than 0.05 were considered significant.

## Results

### TNBS-induced experimental colitis enhanced the autophagy in intestine

**Fig. 1:**
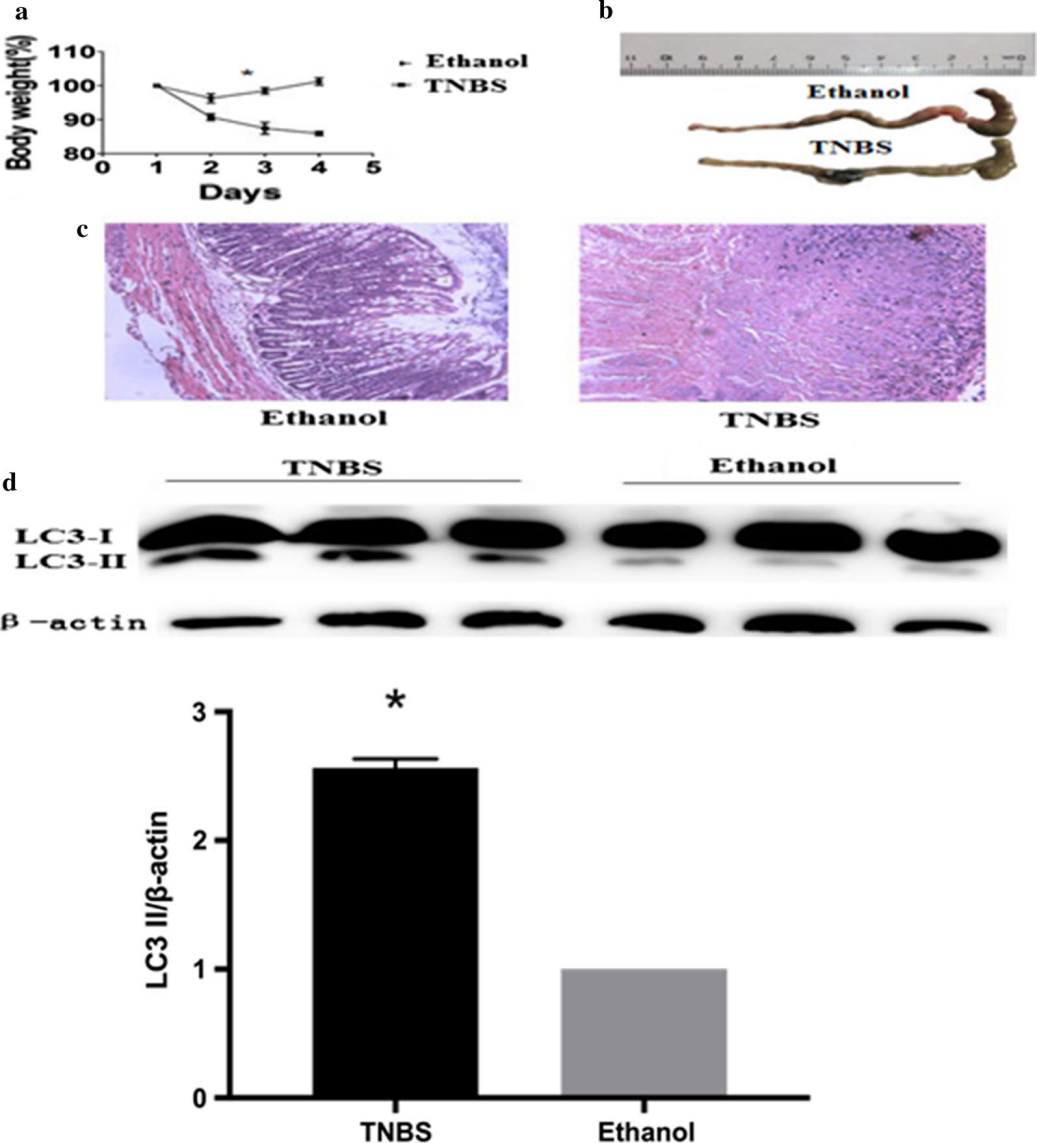
Treatment with 2,4,6-trinitrobenzenesulfonic acid (TNBS) enhanced the autophagy in mice with colitis. BALB/c mice were given 2.5 mg/mouse TNBS (dissolved in 50% ethanol) or the vehicle ethanol intrarectally. All the mice were sacrificed on fourth day. **a** Body weight changes were monitored between zeroth to fourth day in different groups of mice. **b** Colon lengths were measured. **c** Histological analysis (hematoxylin and eosin staining) of mice colons was determined under microscope (original magnification, ×200). **d** Extracts from colons were subjected to LC3 western blotting to evaluate for treatment with TNBS versus the vehicle ethanol. Values were mean ± SD. The asterisk indicates p < 0.05. Data were presented as means + SD of three to six individual mice per group and represent > three independent experimental repeats

We first checked the status of autophagy in TNBS-induced colitis. Treatment with TNBS in mice resulted in a significant loss in body weight on the second day as compared to the vehicle treated mice (Fig. 1a). The mice with colitis had markedly shorter colon and more severe tissue damage as compared to the ethanol treated control mice (Fig. 1b). Compared with the vehicle treatment group, the H&E staining indicated the TNBS group exhibited a loss of normal colon mucous membrane structure, a great number of infiltrating inflammatory cells and extensive ulcerations (Fig. 1c). Notably, the autophagy indicator protein LC3BII expression induced by TNBS treatment in intestine was significantly increased as compared to the ethanol control group (Fig. 1d). These results indicated that TNBS treatment led to activation of the autophagy in intestine with colitis.

### Inhibition of autophagy exacerbated the TNBS-induced experimental colitis

**Fig. 2:**
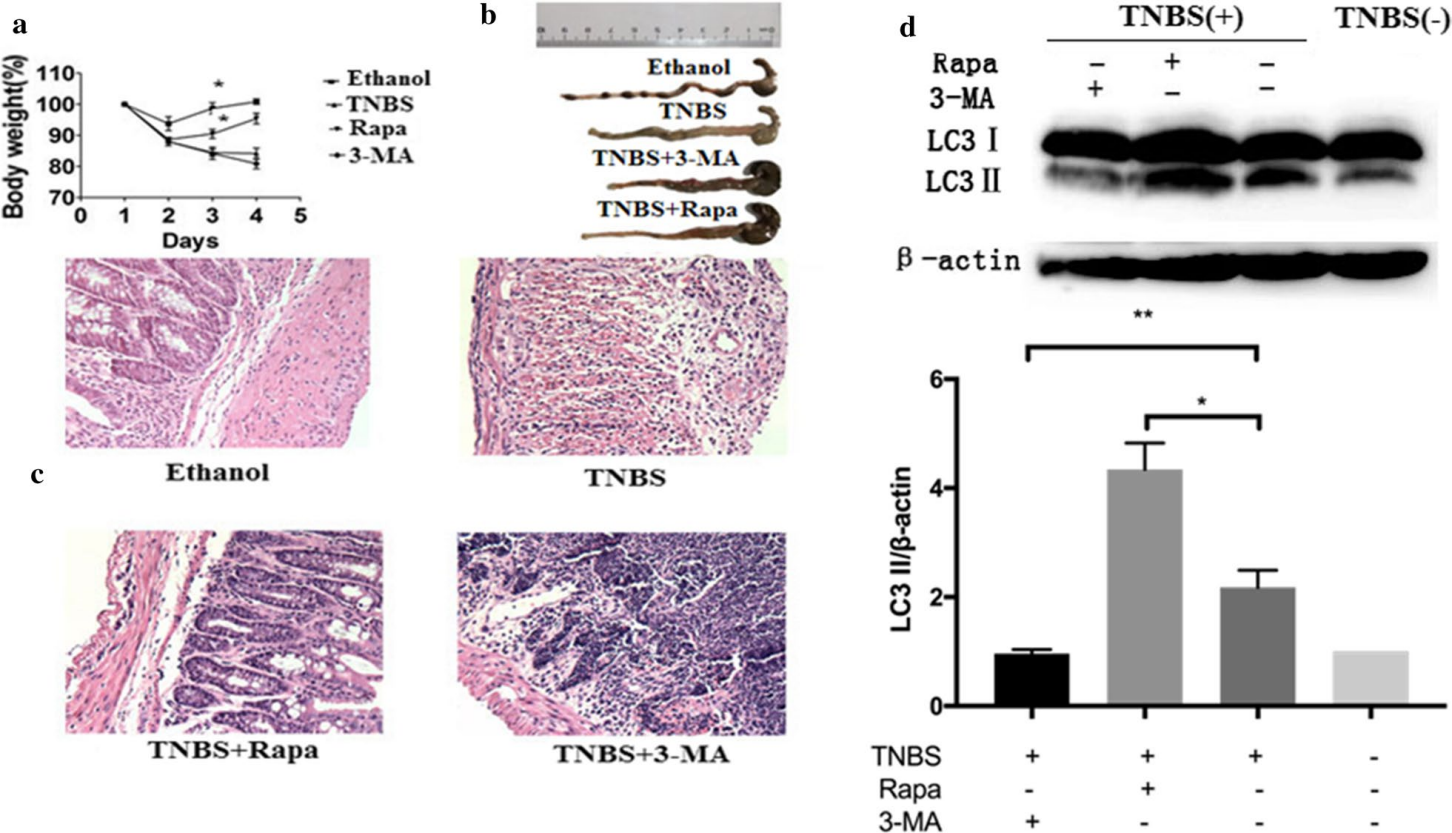
Enhancing of autophagy ameliorates experimental colitis in mice. BALB/c mice were given 2.5 mg/mouse TNBS (dissolved in 50% ethanol), TNBS + rapamycin, TNBS + 3-MA, or the vehicle ethanol intrarectally, respectively. All the mice were sacrificed on fourth day. **a** Body weight changes were monitored between zeroth to fourth day in different groups of mice. **b** Colon lengths were measured. **c** Histological analysis (hematoxylin and eosin staining) of mice colons was determined under microscope (original magnification, ×200). **d** Extracts from colons were subjected to LC3 western blotting to evaluate for treatment with TNBS, TNBS + rapamycin, TNBS + 3-MA versus the vehicle ethanol. Values were mean ± SD. Data were presented as means + SD of three to six individual mice per group and represent > three independent experimental repeats. *p ≤ 0.05, **p ≤ 0.01

We next examined the effects of inhibition of autophagy on the development of TNBS-induced colitis. For this purpose, 3-methyladenine (3-MA),an autophagy inhibitor, and rapamycin, an autophagy-enhancer, were administrated to the mice with TNBS-induced colitis,respectively. The body weight loss of mice with colitis was improved as compared to treatment with TNBS alone group after application of autophagy enhancer, rapamycin (Fig. 2a). In contrast, inhibition of autophagy with 3-MA worsen the loss of body weight of mice with colitis (Fig. 2a). Consistently, the mice treated with TNBS combined with rapamycin displayed longer colon than that of treatment of TNBS alone Fig. 2b). On the contrary, the mice treated with TNBS combined with 3-MA had shorter colon as compared to the mice treated with TNBS alone (Fig. 2b). Pathological analysis indicated that the intestine of mice with colitis treated with TNBS combined with rapamycin exhibited less infiltrates of inflammatory leukocytes than those of the mice treated with TNBS alone. In contrast, the intestine from the mice treated with TNBS combined with 3-MA exhibited a loss of normal colon mucous membrane structure, a great number of infiltrating inflammatory cells, and extensive ulcerations, as compared to TNBS alone group (Fig. 2c). Moreover, Western blotting detected the autophagy-related proteins LC3B1/11 expression in intestines of mice with TNBS-induced colitis. It showed that treatment of the mice with TNBS combined with rapamycin exhibited higher expression of LC3BII than that of the mice treated with TNBS alone. On the contrary, the mice treated with TNBS combined with 3-MA displayed lower expression of LC3BII as compared to the mice treated with TNBS alone (Fig. 2d). Taken together, these results demonstrated that inhibition of autophagy worsens the colitis induced with TNBS in mice.

### IL-33 attenuated the TNBS-induced experimental colitis through enhancing autophagy

**Fig. 3:**
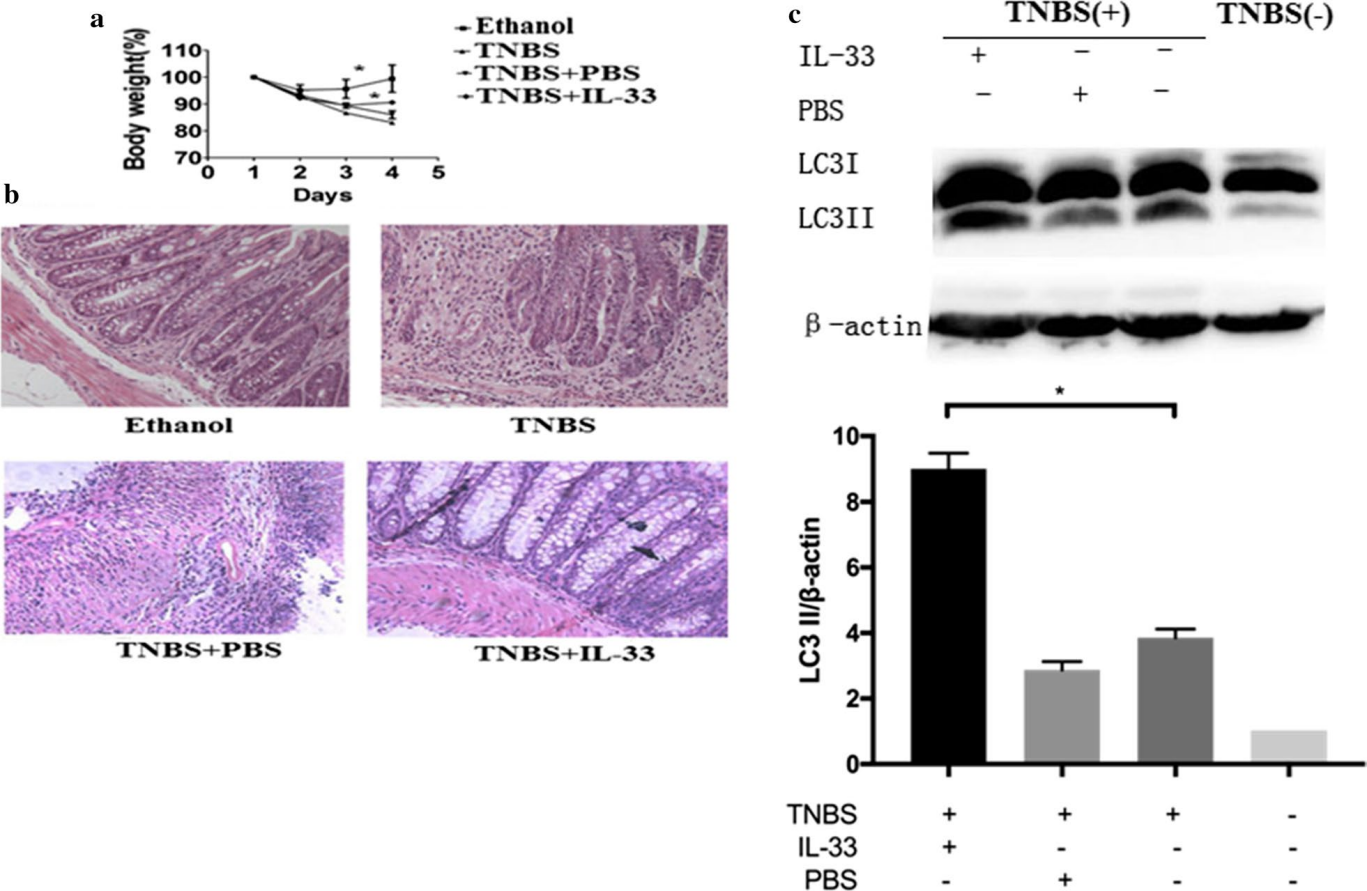
Treatment with IL-33 ameliorates experimental colitis through enhancing autophagy in mice. BALB/c mice were given 2.5 mg/mouse TNBS (dissolved in 50% ethanol), TNBS + PBS, TNBS + IL-33, or the vehicle ethanol intrarectally, respectively. All the mice were sacrificed on fourth day. **a** Body weight changes were monitored between zeroth to fourth day in different groups of mice. **b** Colon lengths were measured. **c** Histological analysis (hematoxylin and eosin staining) of mice colons was determined under microscope (original magnification, ×200). **d** Extracts from colons were subjected to LC3 western blotting to evaluate for treatment with TNBS, TNBS + PBS, TNBS + IL-33 versus the vehicle ethanol. Values were mean ± SD. The asterisk indicates p < 0.05. Data were presented as means + SD of three to six individual mice per group and represent > three independent experimental repeats

We have previously demonstrated that IL-33 ameliorates experimental colitis through promoting Th2/Foxp3^+^ Treg responses in mice [7]. To further exploring the underlying the molecular mechanisms by which IL-33 ameliorates experimental colitis, we treated mice with the vehicle ethanol, TNBS alone, TNBS combined with IL-33, and TNBS combined with PBS control, respectively. As expectedly, administration of IL-33 improved the loss of body weight as compared to TNBS alone or TNBS combined with PBS (Fig. 3a). Consistently, The H&E staining indicated the TNBS group and TNBS combined with PBS group exhibited a loss of normal colon mucous membrane structure, a great number of infiltrating inflammatory cells, and extensive ulcerations, as compared to the vehicle ethanol (Fig. 3b). In contrast, treatment with TNBS combined with IL-33 apparently attenuated the inflammations in intestine, exhibiting less infiltrates of inflammatory leukocytes than those of treated with TNBS (Fig. 3b). Moreover, comparing with treatment with TNBS alone, TNBS combined with IL-33 enhanced the autophagy of colon, evidenced by promoting the conversion of LC3-I to LC3-II (Fig. 3c). These results suggested that IL-33 alleviated the TNBS-induced colitis via promoting the autophagy in intestine.

### TNBS combined with IL-33 promotes the autophagy of macrophages in inflammatory gut tissue

**Fig. 4:**
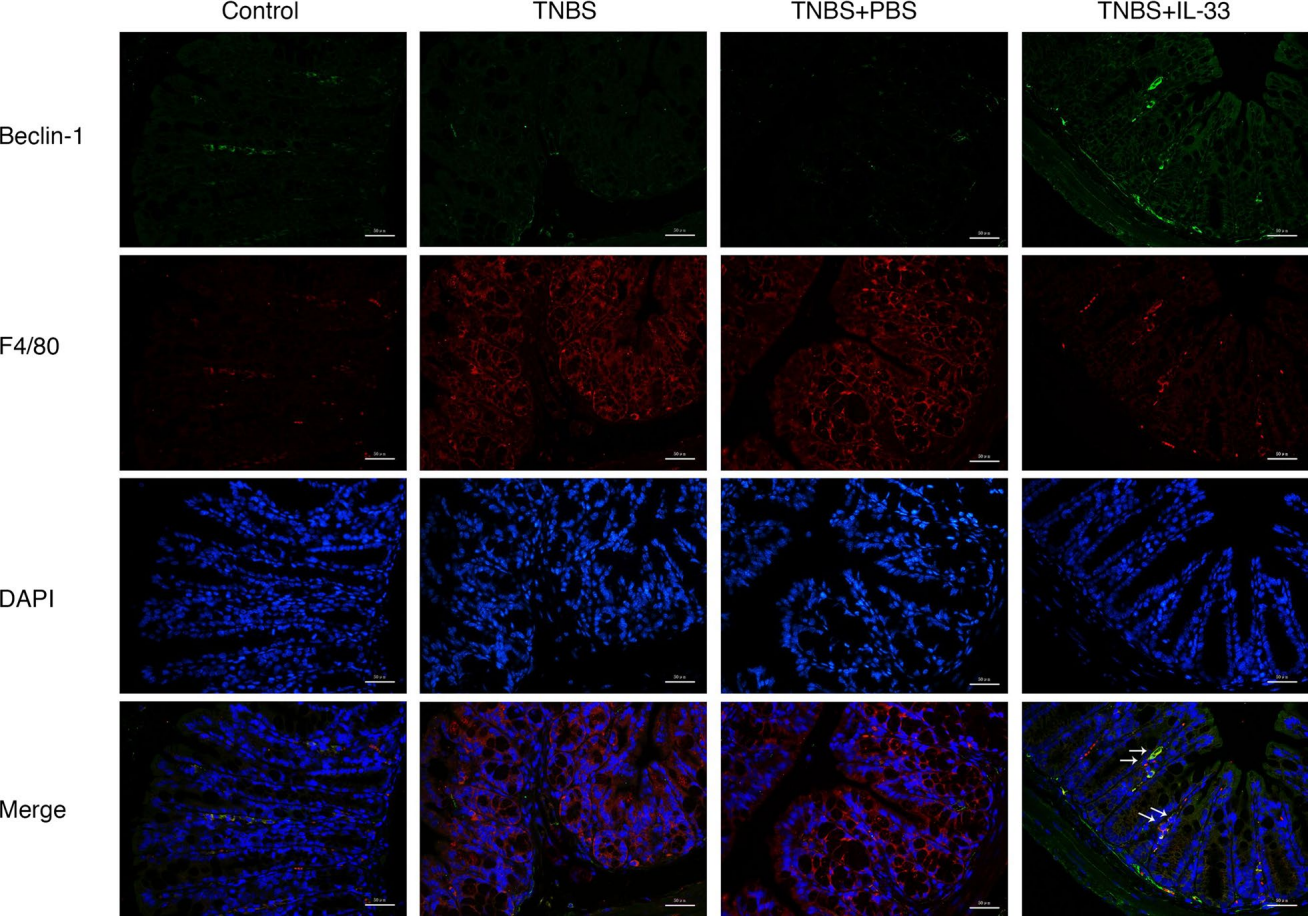
Treatment with IL-33 ameliorates experimental colitis partly through enhancing autophagy of macrophages located in colon tissues. The colon tissues from mice under indicated treatment were co-stained by Beclin-1 (green) and F4/80 (red) immunofluorescent antibody. DAPI (blue) was used to visualize nuclei. Representative microscopic pictures are shown. Magnification, ×200; Scale bar, 50 μm. The experiments were replicated at least twice

To directly examine the activity of macrophages in the inflammatory gut tissue, immunofluorescence staining was performed to detect the status of autophagy of macrophages in the gut tissue. It showed that the autophagy of macrophages in the gut tissue of mice treated with TNBS plus IL-33 was markedly augmented than that of TNBS treatment alone (Fig. 4). This result indicates that IL-33 promotes the autophagy of macrophages in the setting of TNBS-induced experimental colitis.

### Treatment of macrophages with IL-33 enhances autophagy of macrophages

**Fig. 5:**
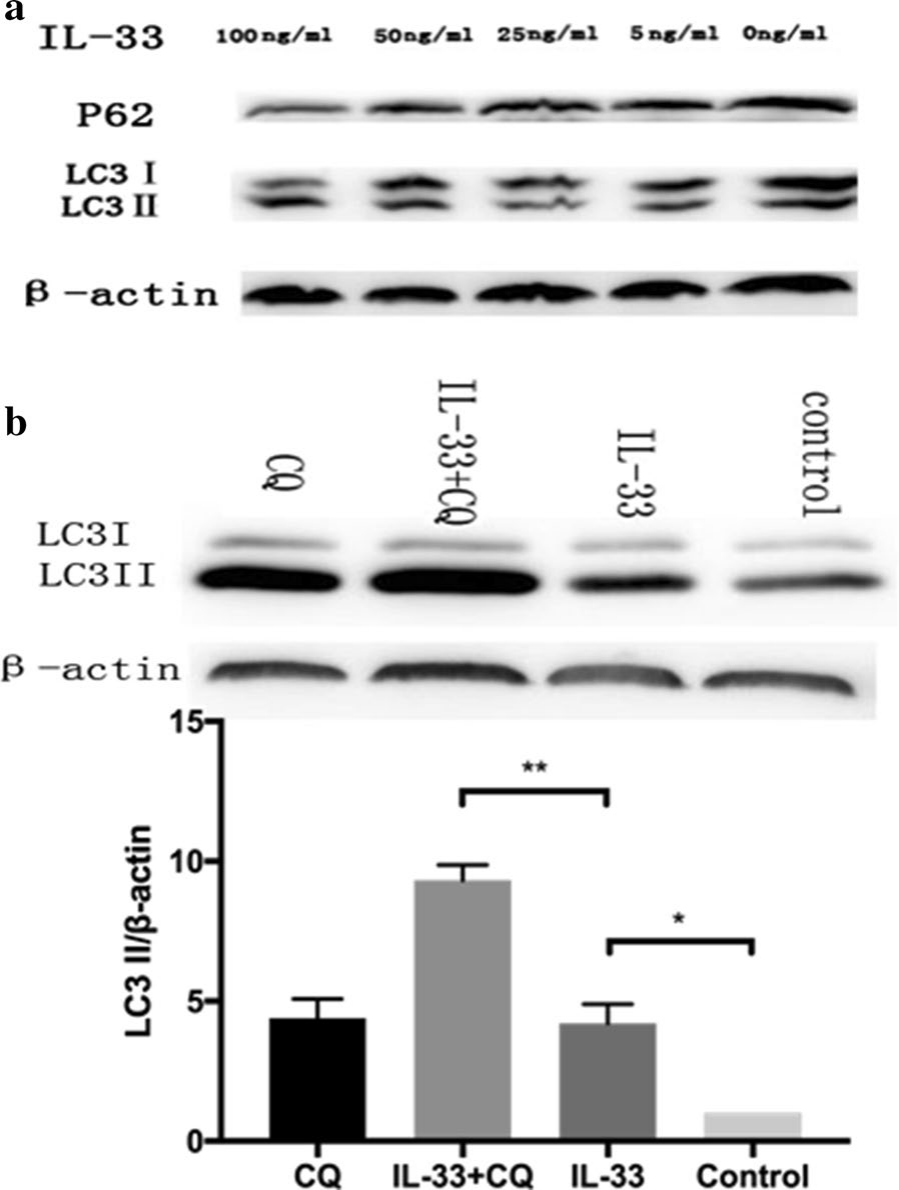
IL-33 enhances the autophagy of macrophages. Bone marrow-derived macrophages were prepared as described in materials and methods. These cells were incubated with different concentrations (100 ng/ml, 50 ng/ml, 25 ng/ml, and 5 ng/ml) of IL-33 for 24 h. Thereafter, the proteins were extracted from these cells and subjected to P62, LC3 and β-actin western blotting to evaluate the autophagy-enhancing effects by IL-33 (**a**). LC3 immunoblots were conducted to evaluate the autophagy induced by IL-33, chloroquine (CQ), and IL-33 + chloroquine (CQ) (**b**). The experiments were repeated three times. *p ≤ 0.05, **p ≤ 0.01

To further confirm the autophagy-enhancing role of IL-33, we conducted the dose–response experiment of IL-33. Treatment with TNBS combined with different doses of IL-33, resulted in a dose-dependent decrease in amounts of p62, a selective autophagy substrate, and a dose-dependent conversion of the non-lipidated form of LC3, LC3-I, to the lipidated, autophagosome-associated form of LC3, LC3-II, in bone marrow-derived macrophages (Fig. 5a). Treatment with 100 ng/ml of IL-33 is an optimal concentration for promoting the autophagy of macrophages, and synergy with chloroquine (CQ), a recognized antophagy-enhancer, to promote the conversion of LC3-I to LC3-II (Fig. 5b). Collectively, these results demonstrated that IL-33 ameliorates experimental colitis through, at least in part, regulating autophagy in mice.

### IL-33 enhanced the autophagy via regulating TLR4 signaling pathway

**Fig. 6:**
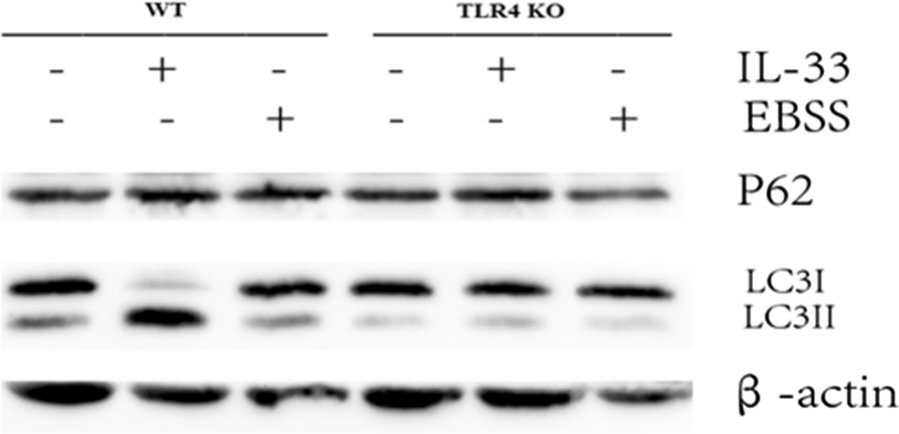
IL-33 promotes the autophagy of macrophages via regulating the TLR4 signaling pathway. Bone marrow-derived macrophages were prepared from wild-type (WT) mouse and TLR4 knockout (KO) mouse, respectively. Macrophages derived from WT or TLR4KO were treated with IL-33 or starvation medium EBSS respectively, and cell lysates were immunoblotted to detect P62, LC3I, LC3II and β-actin. The experiments were repeated at least two times

Next, to tackle the underlying mechanism of IL-33 enhancing the autophagy, we compared the effects of IL-33 on the autophagy of macrophages derived from TLR4 knockout mice and wild-type control mice. As shown in Fig. 6, both IL-33 and cellular starvation (EBSS treatment) enhanced the autophagy of macrophages, as evidenced by promoting the conversion of LC3-I to LC3-II. Furthermore, deficiency of TLR4 receptor resulted in disappeared of the autophagy activation by treatment with IL-33 or EBSS (Fig. 6). The results demonstrated that TLR4 signaling pathway is necessary for IL-33 to activate the autophagy of macrophages.

### Deficiency of IL-33 of bone marrow-derived macrophages displayed dramatically change of autophagy induced by rapamycin treatment

**Fig. 7:**
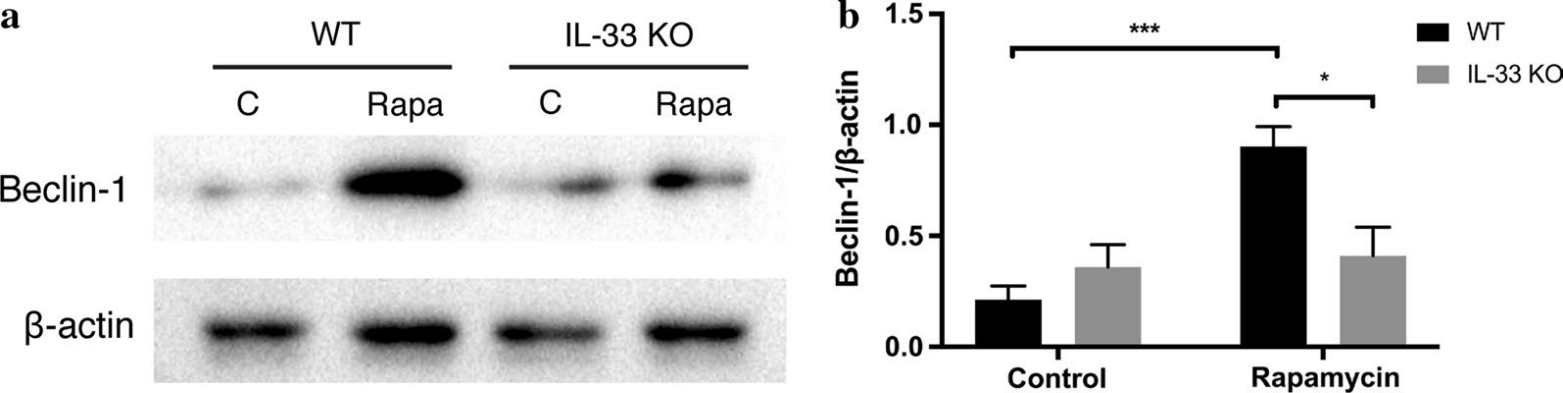
Deficiency of IL-33 impaired the sensitivity of macrophages to rapamycin-induced autophagy. Bone marrow-derived macrophages were generated from IL-33 gene knockout mice and wild type control mice, respectively. **a** Western blotting against becklin-1 was conduced to determine the role of IL-33 on autophagy induced by rapamycin treatment. **b** Bar graph showing the quantification the western blot bands. The experiment was repeated twice, *p ≤ 0.05, ***p ≤ 0.001

Finally, to further confirm the above results, IL-33-deficient macrophages derived from IL-33 gene knockout mice were used to examine the regulatory role of IL-33 on autophagy. We observed that macrophages with IL-33 gene (wild type) exhibited enhanced the sensitivity to rapamycin-induced the autophagy (Becklin-1 as an indicator of autophagy) as compared to IL-33-deficient macrophages (Fig. 7). This result indicated that IL-33 participated in the regulation of autophagy of macrophages.

## Discussion

In the present study, we investigated the effects and mechanisms of IL-33 on experimental colitis induced by TNBS in mouse. Consistent with our previous observation that treatment with IL-33 ameliorated the severity of colitis, improved the loss of animal body weight, relieved the activity of illness, and decreased the infiltration of inflammatory leukocytes. A novel finding is that IL-33 ameliorates experimental colitis through enhancing autophagy in mice.

There is a growing interest in the role of autophagy in normal health and in various disease states [15–18]. Modulation of autophagy is a novel therapeutic strategy for diseases including HIV [19]. What are the possible mechanisms by which IL-33 enhancing autophagy alleviate TNBS-induced experimental colitis? IL-33 localizes to intestinal epithelial cells, and its full-length form is released upon epithelial damage, acting as an “alarmin” and triggering wound healing at the mucosa [20]. Increased mucosal IL-33 in human UC and murine colitis may be a homeostatic response to limit inflammation, potentially through effects on epithelial barrier function [21]. We can hypothesize that that activation of the autophagy of intestinal epithelial cells confers these cells to be resistant to inflammatory insult elicited by TNBS treatment. Furthermore, macrophage is one of the largest population of macrophages resides in the gastrointestinal tract [22]. IL-33 is expressed by CD14^+^ macrophages [23], and plays a therapeutic role in autoimmune CNS disease by switching a predominantly pathogenic Th17/Th1 response to Th2 activity, and by polarization of anti-inflammatory M2 macrophage [24]. Likewise, the autophagy-enhanced macrophages by IL-33 could be more polarized to M2 against the TNBS-induced Th1 inflammatory injury in the setting of colitis.

Cytokine modulation of autophagy is increasingly recognized in disease pathogenesis, and current concepts suggest that type 1 cytokines activate autophagy, whereas type 2 cytokines are inhibitory. However, this paradigm is poorly characterized in tissue cells, including sentinel epithelial cells that regulate the immune response. In particular, the type 2 cytokine IL-13 (interleukin 13) drives the formation of airway goblet cells that secrete excess mucus as a characteristic feature of airway disease. It has been shown that autophagy is essential for airway mucus secretion in a type 2, IL-13-dependent immune disease process and the regulation of autophagy by Th2 cytokines is cell-context dependent [25]. In support to our notion, deletion of the IL-33 gene impaired normal disposal of atretic follicles, resulting in massive accumulations of tissue wastes abundant with aging-related catabolic wastes such as lipofuscin. Accumulation of tissue wastes in IL-33(−/−) mice, in turn, accelerated ovarian aging and functional decline. Thus, their reproductive life span was shortened to two-thirds of that for IL-33(±) littermates. IL-33 orchestrated disposal mechanism through regulation of autophagy in degenerating tissues and macrophage migration into the tissues [26]. However, IL-33 treatment apparently suppressed the expression of pro-inflammation cytokines IL-1β and TNF-α, evidently increased Bcl-2 but decreased cleaved-caspase-3, and obviously decreased the levels of autophagy-associated proteins LC3-II and Beclin-1 but maintained P62 at high level after ICH. On the contrary, treatment with sST2, a decoy receptor of IL-33, exacerbated ICH-induced brain damage and neurological dysfunction by promoting apoptosis, and enhancing autophagic activity [27]. This observation indicates that the regulation of autophagy by IL-33 is also cell-context dependent.

Besides LC3, levels of other autophagy substrates can be used to monitor autophagic flux. p62 (also known as SQSTM1/sequestome 1) is selectively incorporated into autophagosomes through direct binding to LC3 and is efficiently degraded by autophagy [28]. To evaluate the modulation of autophagy by IL-33, we observed that LC3 expression as an indicator of autophagy is better than that of p62 because LC3 is more sensitive to the treatment with IL-33 (Fig. 6). Moreover, there appears a requirement for the concentration of IL-33 to modulate the autophagy of macrophages (Fig. 5).

It has previously been demonstrated that IL-33 increases the expression of the LPS receptor components MD2 (myeloid differentiation protein 2) and TLR-4, the soluble form of CD14 and the MyD88 adaptor molecule. In addition, IL-33 pretreatment of macrophages enhances the cytokine response to TLR-2 but not to TLR-3 ligands. Thus, IL-33 treatment preferentially affects the MyD88-dependent pathway activated by the TLR [29]. In line with these findings, we show that IL-33 also activates the autophagy of macrophages through regulating TLR4 signaling pathway, because TLR4 deficiency abolishes the enhancement of the autophagy of macrophages by IL-33 treatment. Nevertheless,further studies will be required to delineate the specific molecules as well as cells of intestine modulated by IL-33 in the setting of TNBS-induced experimental colitis in mice.

In conclusion, we presented strong evidences to support that IL-33 regulating autophagy plays a role, at least in part, in alleviating the inflammation in the context of TNBS-induced experimental colitis. Therefore, modulation of the autophagy in intestine might have therapeutic implications in colitis.
